# Clinical analysis and artificial intelligence survival prediction of serous ovarian cancer based on preoperative circulating leukocytes

**DOI:** 10.1186/s13048-022-00994-2

**Published:** 2022-05-24

**Authors:** Ying Feng, Zhixiang Wang, Ran Cui, Meizhu Xiao, Huiqiao Gao, Huimin Bai, Bert Delvoux, Zhen Zhang, Andre Dekker, Andrea Romano, Shuzhen Wang, Alberto Traverso, Chongdong Liu, Zhenyu Zhang

**Affiliations:** 1 Department of Obstetrics and Gynecology, Beijing Chao-Yang Hospital, Capital Medical University, Beijing, China; 2 Department of Obstetrics and Gynecology, GROW-School for Oncology and Developmental Biology, Maastricht University Medical Centre, Maastricht, The Netherlands; 3 Department of Radiation Oncology (Maastro), GROW-School for Oncology, Maastricht University Medical Centre+, Maastricht, The Netherlands; 4 Department of Obstetrics and Gynecology, Peking University First Hospital, Beijing, China

**Keywords:** Machine learning, Leukocytes, Serous ovarian cancer, Recurrence, Survival, And prediction

## Abstract

Circulating leukocytes are an important part of the immune system. The aim of this work is to explore the role of preoperative circulating leukocytes in serous ovarian carcinoma and investigate whether they can be used to predict survival prognosis. Routine blood test results and clinical information of patients with serous ovarian carcinoma were retrospectively collected. And to predict survival according to the blood routine test result the decision tree method was applied to build a machine learning model.

The results showed that the number of preoperative white blood cells (*p* = 0.022), monocytes (*p* < 0.001), lymphocytes (*p* < 0.001), neutrophils (*p* < 0.001), and eosinophils (*p* < 0.001) and the monocyte to lymphocyte (MO/LY) ratio in the serous ovarian cancer group were significantly different from those in the control group. These factors also showed a correlation with other clinicopathological characteristics. The MO/LY was the root node of the decision tree, and the predictive AUC for survival was 0.69. The features involved in the decision tree were the MO/LY, differentiation status, CA125 level, neutrophils (NE,) ascites cytology, LY% and age.

In conclusion, the number and percentage of preoperative leukocytes in patients with ovarian cancer is changed significantly compared to those in the normal control group, as well as the MO/LY. A decision tree was built to predict the survival of patients with serous ovarian cancer based on the CA125 level, white blood cell (WBC) count, presence of lymph node metastasis (LNM), MO count, the MO/LY ratio, differentiation status, stage, LY%, ascites cytology, and age.

## Background

Ovarian carcinoma is the 5th leading cause of cancer-related deaths among women and the deadliest disease among gynecological malignancies [1, 2]. Statistics from the United States show that the number of new cases of ovarian carcinoma in 2021 will be 22,530, and the number of deaths per year is estimated at approximately 13,770 [1]. Ovarian cancer usually has a poor prognosis because many patients already present with advanced metastatic stages before diagnosis [2, 3]. The 1-year survival rate is approximately 72%, the 5-year survival rate is 48%, and the 10-year survival rate is approximately 35% [2, 4, 5]. Serous carcinoma accounts for 75% of all ovarian cancers and is the most common pathological type [3, 6]. Therefore, it is worthwhile to preoperatively predict the survival of serous ovarian carcinoma using clinicopathological features to guide decisions regarding surgery and postsurgical care.

Some reports have indicated that the interaction between ovarian cancer and the immune system may affect tumor growth and progression [7, 8]. There is also some evidence that the inflammatory process caused by pelvic inflammatory disease may be associated with ovarian cancer [9]. Regarding the tumor evasion mechanism, tumor cells modulate the immune response for their benefit; tumor cells secrete specific cytokines that recruit and stimulate the production of myeloid-derived suppressor cells (MDSCs). They also produce TGF-β and IL-10 and inhibit T lymphocytes, macrophages and dendritic cells to create an immunosuppressive tumor microenvironment [10–12].

Due to the prominent role of the immune system in ovarian cancer, preoperative immune and inflammatory features may be suitable prognostic biomarkers. One promising characteristic is the leukocyte count.

Leukocytes, also called white blood cells (WBCs), are immune cells involved in protecting the body from disease and pathogens [13–15]. WBCs are distributed throughout the body, including the blood system and lymphatic system. WBCs account for approximately 1% of the total blood volume of healthy adults. There are five main subtypes of leukocytes: lymphocytes, monocytes, neutrophils, eosinophils, and basophils. They have a great impact on health because human immunity is based on the presence of and balance among these cell types. When an immune response occurs, as in the case of cancer, the number of WBCs will change accordingly [7, 16–18].

Higher monocyte counts were reported to be associated with a poor prognosis in patients with endometrial cancer [19]. The lymphocyte-to-monocyte ratio (LMR) in patients with epithelial ovarian cancer (EOC) and those with benign ovarian masses is significantly different [16]. The lymphocyte-to-monocyte ratio (LMR) has been significantly associated with the stage of EOC [20] and can provide prognostic information [21]. The monocyte-to-lymphocyte ratio has also been shown to predict shorter overall survival (OS) and progression-free survival (PFS) in EOC patients [22]. Therefore, we also paid attention to the monocyte-to-lymphocyte ratio in patients with serous ovarian cancer.

In this study, we aimed to explore the potential role of WBCs as prognostic biomarkers. Our primary objective is to investigate whether the number and proportion of circulating leukocytes in patients with serous ovarian carcinoma are different from those in normal controls (uterine prolapse patients). We also aimed to determine their association with clinicopathological characteristics, survival, and prognosis. As a secondary objective, we explored whether the test of preoperative circulating leukocytes can be used to predict the survival of ovarian serous carcinoma. To this end, machine learning in artificial intelligence (AI) [23], which is widely used in various medical fields, such as anatomy and brain-machine interfaces [24], is used to develop algorithms to predict the survival of patients with serous ovarian cancer.

## Methods

### Study subject

This study retrospectively analyzed patients with ovarian serous carcinoma who were initially treated at the Department of Obstetrics and Gynecology at Beijing Chaoyang Hospital, Capital Medical University, from July 2009 to December 2018. The case inclusion criteria were as follows: (1) surgical treatment performed at Beijing Chaoyang Hospital, (2) confirmation of ovarian serous carcinoma (serous cystadenocarcinoma or high-grade serous cystadenocarcinoma) by postoperative pathology, (3) standard platinum-based chemotherapy after the first tumor reduction surgery, and (4) complete preoperative routine blood and clinical data. The exclusion criteria were as follows: (1) presence of other types of benign and/or malignant ovarian tumors, (2) presence of primary malignant tumors of other organs, (3) no standardized chemotherapy after the first tumor reduction operation, and (4) incomplete routine blood and clinical data. The obtained data included age, BMI, childbirth history, menopause, neoadjuvant chemotherapy, surgical satisfaction, differentiation, stage based on the 2014 International Federation of Gynecology and Obstetrics (FIGO) staging system [25], ascites cytology, lymph node metastasis (LNM), recurrence, which is defined as the time from the first cytoreductive surgery to the time of ovarian cancer recurrence, death of disease (DOD) that is defined as the date from the first cytoreductive surgery to the date of the patient's death due to ovarian cancer, preoperative leukocyte count and proportion (within 90 days before the operation).

Recurrence was defined as the time from the first cytoreductive surgery to the time of ovarian cancer recurrence; death was defined as the date from the first cytoreductive surgery to the date of the patient's death due to ovarian cancer. The “normal”/control group selected and consisted of patients of a similar age who were diagnosed with uterine prolapse. The results of preoperative routine blood tests for these patients were also collected. Ethics approval for this research was provided by the Beijing Chaoyang Hospital, Capital Medical University (approval number 2021-ke-205, study number 2012DRF30490).

### Statistical analysis

Statistical analysis of the clinical data was performed with SPSS (version 23.0, IBM). Continuous data are expressed as the median and compared using the Mann–Whitney U test. Total/differential leukocytes were divided into two groups according to the median value. Kaplan–Meier survival curves were then performed to compare overall survival (OS), which is defined as the time from the date of surgery to death (due to serous ovarian cancer), and progression-free survival (PFS), which is defined as the time from the date of surgery to recurrence, between the two groups. Significance was tested using the log-rank test, where these patients (5 patients) who had different first chemotherapy regimen were excluded. The three-dimensional (3D) histograms with three variables were constructed with Python 3.8. The significance was set at a two-sided *p* value < 0.05.

### Machine learning

**Fig. 1:**
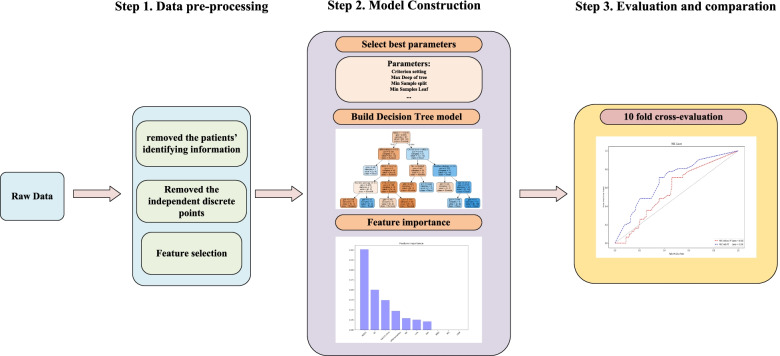
Flowchart for decision tree prediction. To build a machine learning model, first, data pre-processing is required. Second, select best parameters and build the model. Third, evaluation and compare the model performance

For survival prediction, we choose the machine learning-based decision tree algorithm. We divided the method into several steps, as shown in the flowchart (Fig. 1). We implemented the algorithm in Python 3.8 and the scikit-learn 0.24 package. In the preprocessing part, we first removed the patients’ identifying information. Second, we analyzed the distribution and removed the independent discrete points that were out of the value range of 5–95%. Third, we selected the features, such as stage, grade of differentiation and LNM, according to the National Comprehensive Cancer Network (NCCN) guideline [26]. Then, using tenfold cross-validation, we separated the data into two parts: 90% of the data was used for training and 10% of the data was used for testing. Imbalanced datasets are often handled well by decision tree classifiers [27], so we built a decision tree model and trained the model. Finally, to ensure the stability of the model, we used tenfold cross-validation to train and test the model. To reduce the influence of imbalanced data, we used the synthetic minority oversampling technique (SMOTE) method to oversample the training set, which is an improved scheme based on a random oversampling algorithm [28]. To prove the role of circulating leukocytes in survival prediction, we performed comparisons with the same model trained by the features without circulating leukocytes.

For the decision tree learning process, these patients (93 patients) who had same first chemotherapy regimen were included. The optimal feature was selected recursively, and the training data were segmented according to the feature so that each subdataset had the best classification process. This process corresponded to the division of the feature space and the construction of the decision tree. First, the root node was constructed, and all training data were placed in the root node. An optimal feature was chosen, and the training dataset was divided into subsets according to this feature so that each subset had the best classification under the current conditions. If these subsets could be relatively correctly classified, then the leaf nodes were constructed, and these subsets were divided into the corresponding leaf nodes; if there were still subsets that could not be relatively correctly classified, then these subsets selected the new optimal feature, continued to divide it, and constructed the corresponding node. This process proceeded recursively until all the training data subsets were basically correctly classified or there were no suitable features. Finally, each subset was assigned to the leaf nodes.

## Results

### Patient clinicopathological characteristics and preoperative circulating leukocytes

A total of 98 patients with ovarian serous carcinoma who were initially treated at the Department of Obstetrics and Gynecology at Beijing Chaoyang Hospital, Capital Medical University, from July 2009 to December 2018 were included in the analysis according to the inclusion and exclusion criteria. The first chemotherapy regimen after the first surgery for all selected patients was platinum-based treatment (93 patients received 6–8 cycles of paclitaxel and cisplatin (PT), 3 patients received 8 cycles of cisplatin + adriamycin + cyclophosphamide (PAC), 1 patient received 8 cycles of paclitaxel and carboplatin, and 1 patient received 4 cycles of PT and 2 cycles of cisplatin + etoposide + ifosfamide (PEI)). The average age was 57 years old, and the mean BMI was 24.3. The pathological results revealed that 88.60% of the patients had poorly differentiated tumors (G3), 79.60% had stage III disease, 66.3% had positive ascites cytology, and 43.2% had LNM. The recurrence and mortality rates were 55.3% and 29.7%, respectively, at the time of follow-up (28 July 2019).

**Fig. 2:**
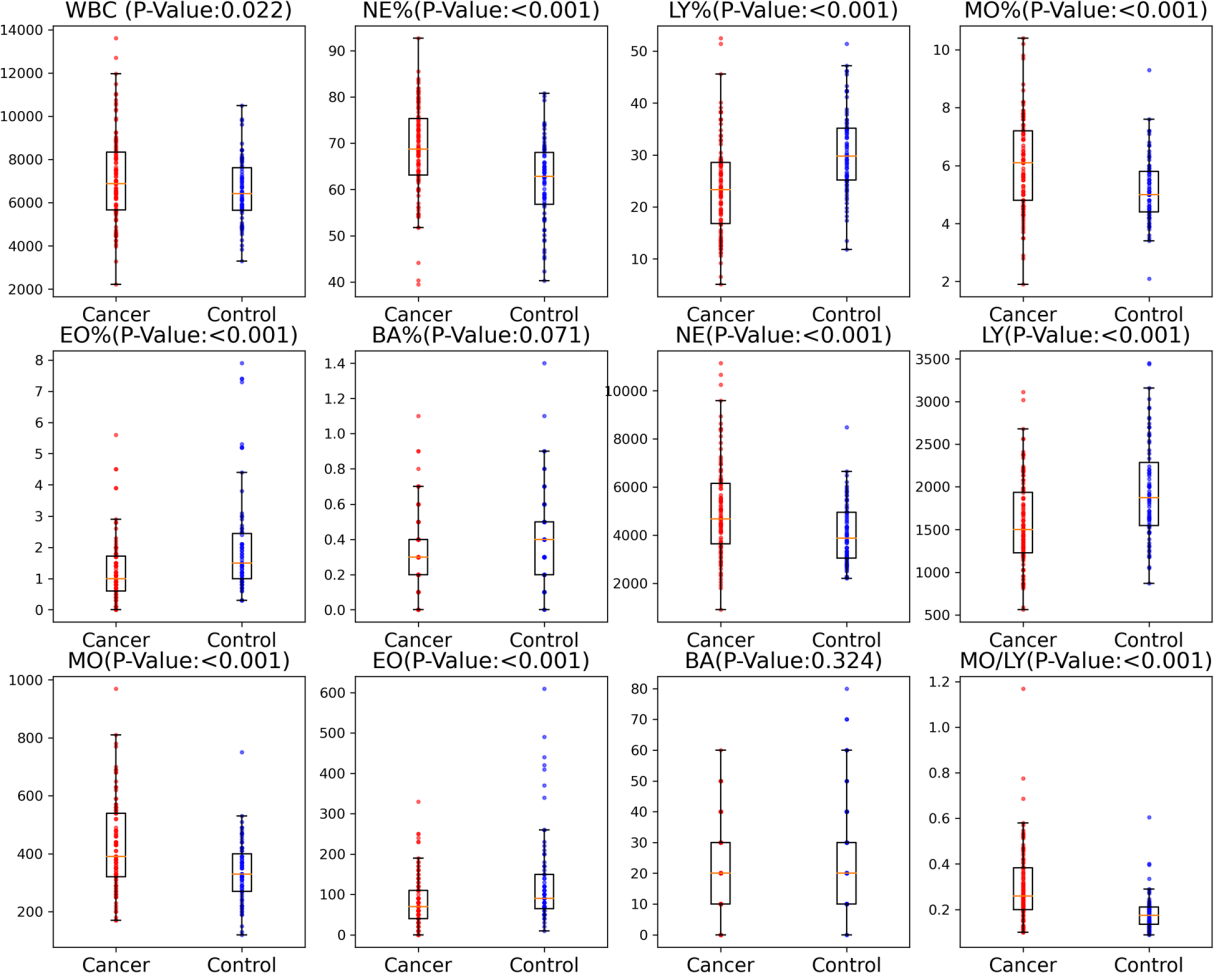
Boxplot distribution diagram of white blood cells. The mean ± SEM of preoperative white blood cells were compared between control and serous ovarian cancer samples using a boxplot. Preoperative WBC, NE, NE%,MO, MO% and MO/LY in ovarian serous carcinoma patients (*n* = 98) were significantly higher than those in control group (*n* = 75), while LY, LY%, EO and EO% were significantly lower than those in control group. BA and BA% showed no difference between the two groups

The preoperative monocyte count and proportion in the serous ovarian cancer group (98 patients) were significantly higher than those in the control group (75 patients, *p* < 0.001 and *p* < 0.001, respectively, Table 1 and Fig. 2). The monocyte-to-lymphocyte (MO/LY) ratio in the serous ovarian cancer group was also significantly higher than that in the normal control group (p < 0.001). The number of white blood cells (WBCs, *p* = 0.022), lymphocytes (LYs, *p* < 0.001), neutrophils (NEs, *p* < 0.001), and eosinophils (EOs, *p* < 0.001) were also significantly different between the serous ovarian cancer group and the normal control group.

**Table 1:** The comparison of preoperative blood counts between serous ovarian cancer group and control normal group

Blood routine	Median		*P*-Value
	Serous ovarian cancer (*N* = 98)	Control (*N* = 75)	
WBC 10^6^/L	7000	6420	0.022
NE 10^6^/L	4710	3880	< 0.001
LY 10^6^/L	1510	1870	< 0.001
MO 10^6^/L	390	330	< 0.001
EO 10^6^/L	70	90	< 0.001
BA 10^6^/L	20	20	0.324
NE%	68.9	62.8	< 0.001
LY%	23.2	29.8	< 0.001
MO%	6.1	5	< 0.001
EO%	1	1.5	< 0.001
BA%	0.3	0.4	0.071
MO/LY	0.2592	0.1746	< 0.001

*WBC* white blood cells, *NE* neutrophils, *LY* lymphocytes, *MO* monocytes, *EO* eosinophil, *BA* basophils, *MO/LY* the ratio of monocytes to lymphocytes

As shown in Table 2, the percentage of monocytes showed significant differences across the different disease stages (*p* = 0.046); the more advanced the stage was, the higher the average percentage. The monocyte counts also showed similar results, with patients with LNM having more monocytes (*p* = 0.05). The MO/LY ratio showed significant differences according to differentiation status (*p* = 0.029), stage (*p* = 0.007), LNM (*p* = 0.025), and recurrence (*p* = 0.036), with a higher ratio indicating a worse result, similar to the results for CA125. In addition, the number of NE (*p* = 0.049) and BA (*p* = 0.011) and the percentage of LY (LY%, *p* = 0.036) affected LNM. LY% (*p* = 0.048 and *p* = 0.015, respectively) and NE% (*p* = 0.027 and *p* = 0.028, respectively) were significantly correlated with positive ascites cytology and recurrence.

**Table 2:** The relationship between preoperative blood counts and clinicopathological features in patients with serous ovarian cancer

Characteristics (*n* = 98)	WBC 10^6^/L	NE 10^6^/L	LY 10^6^/L	MO 10^6^/L	EO 10^6^/L	BA 10^6^/L	NE %	LY %	MO %	EO %	BA %	MO/LY	CA125 U/ml
Stage	*p* = 0.554	*p* = 0.260	*p* = 0.239	*p* = 0.050	*p* = 0.196	*p* = 0.053	*p* = 0.152	*p* = 0.054	*p* = 0.338	*p* = 0.347	*p* = 0.169	*p* = 0.026	*p* = 0.002
I + II	6430.00	4380.00	2040.00	350.00	70.00	20.00	66.30	27.80	5.50	1.00	0.20	0.19	52.60
III + IV	7000.00	4770.00	1505.00	430.00	75.00	20.00	69.25	22.70	6.10	1.05	0.30	0.27	847.40
Differentiation	*p* = 0.844	*p* = 0.742	*p* = 0.242	*p* = 0.134	*p* = 0.175	*p* = 0.844	*p* = 0.494	*p* = 0.261	*p* = 0.061	*p* = 0.123	*p* = 0.904	*p* = 0.028	*p* = 0.679
Low (G1)	6880.00	4700.00	1490.00	420.00	70.00	20.00	69.05	23.00	6.15	0.90	0.30	0.27	710.60
High (G2 + G3)	7030.00	5540.00	1790.00	340.00	80.00	20.00	68.40	23.90	5.10	1.40	0.30	0.20	457.80
Ascites	*p* = 0.469	*p* = 1.00	*p* = 0.046	*p* = 0.077	*p* = 0.135	*p* = 0.852	*p* = 0.076	*p* = 0.111	*p* = *0*.118	*p* = 0.232	*p* = 0.752	*p* = 0.697	*p* < 0.001
**-**	7540.00	5050.00	2080.00	520.00	100.00	20.00	65.40	26.30	6.30	1.40	0.30	0.25	71.74
+	6850.00	4680.00	1480.00	390.00	70.00	20.00	69.40	22.90	6.10	0.90	0.30	0.26	1118.50
Ascites cytology	*p* = 0.272	*p* = 0.076	*p* = 0.166	*p* = 0.585	*p* = 0.475	*p* = 0.813	*p* = 0.027	*p* = 0.048	*p* = 0.724	*p* = 0.373	*p* = 0.727	*p* = 0.201	*p* = 0.005
-	6465.00	4435.00	1530.00	385.00	85.00	20.00	66.20	26.65	5.70	1.25	0.30	0.23	320.10
+	7000.00	4880	1550.00	430.00	70.00	20.00	70.45	23.00	5.95	1.00	0.30	0.26	1216.59
LNM	*p* = 0.111	*p* = 0.049	*p* = 0.250	*p* = 0.050	*p* = 0.286	*p* = 0.011	*p* = 0.09	*p* = 0.036	*p* = 0.439	*p* = 0.547	*p* = 0.083	*p* = 0.025	*p* = 0.027
-	6470.00	4450.00	1730.00	380.00	70.00	20.00	67.80	25.70	5.80	1.00	0.30	0.24	417.80
+	7330.00	5210.00	1550.00	440.00	80.00	30.00	70.60	20.60	6.10	1.10	0.40	0.30	1094.00
Recurrence	*p* = 0.380	*p* = 0.152	*p* = 0.100	*p* = 0.371	*p* = 0.846	*p* = 0.390	*p* = 0.028	*p* = 0.015	*p* = 0.988	*p* = 0.540	*p* = 0.301	*p* = 0.036	*p* = 0.001
-	6775.00	4465.00	1640.00	390.00	80.00	20.00	65.50	26.50	5.65	1.15	0.30	0.22	272.35
+	7210.00	5050.00	1450.00	430.00	70.00	20.00	70.60	22.00	6.10	1.00	0.30	0.27	1218.00
Dead of disease	*p* = 0.958	*p* = 0.838	*p* = 0.239	*p* = 0.791	*p* = 0.446	*p* = 0.536	*p* = 0.330	*p* = 0.239	*p* = 0.728	*p* = 0.408	*p* = 0.452	*p* = 0.152	*p* = 0.056
-	6955.00	4685.00	1560.00	390.00	80.00	20.00	68.50	23.85	5.70	1.10	0.30	0.25	552.55
+	7000.00	4990.00	1465.00	435.00	60.00	20.00	69.05	22.10	6.25	0.90	0.30	0.29	1216.59

*WBC* white blood cells, *NE* neutrophils, *LY* lymphocytes, *MO* monocytes, *EO* eosinophil, *BA* basophils, *MO/LY* ratio of monocytes to lymphocytes, *LNM* Lymph node metastasis

### Survival analysis based on preoperative circulating leukocytes

**Fig. 3:**
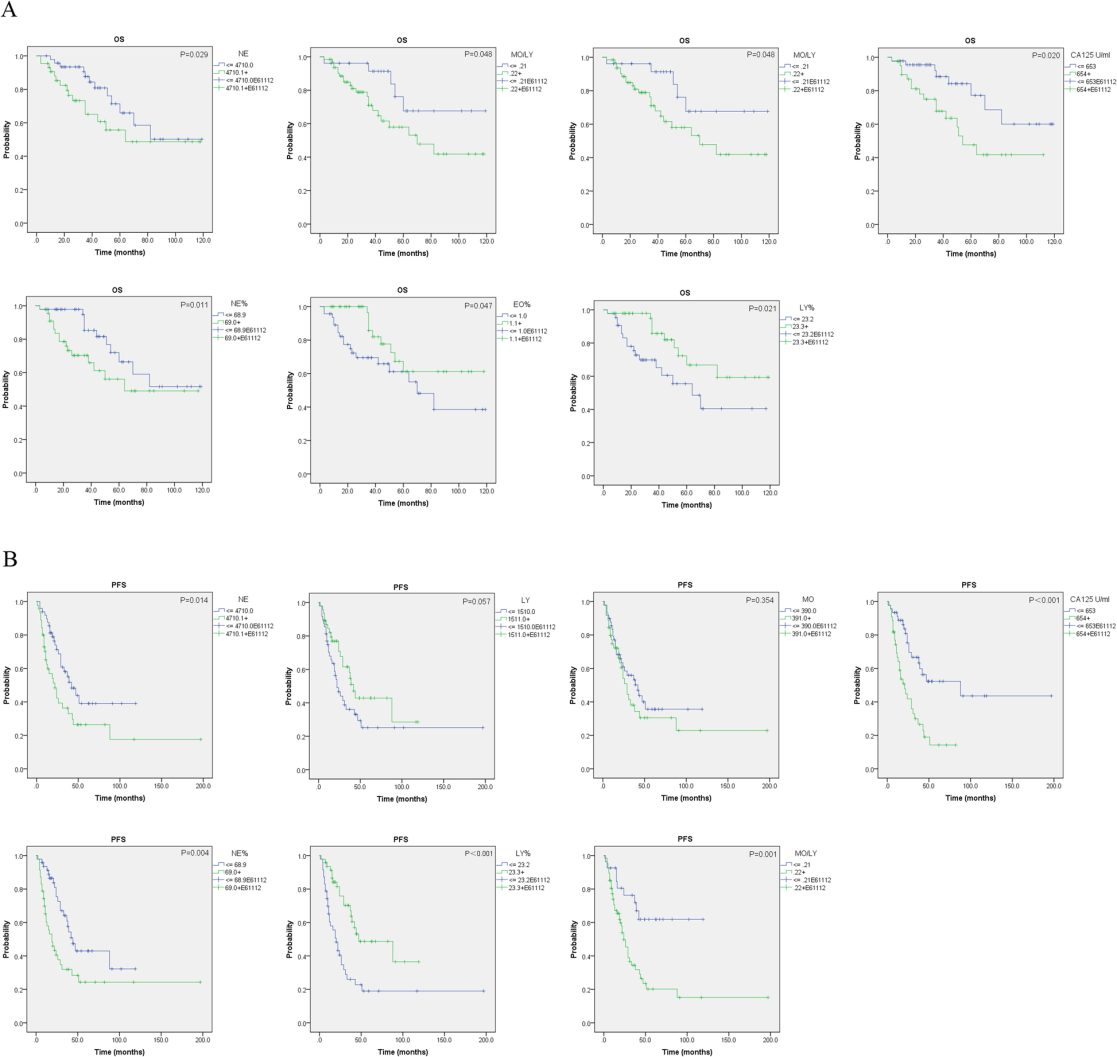
The prognostic value of preoperative blood counts in serous ovarian cancer. The Kaplan–Meier survival curves with the log-rank test were performed and compared between control and serous ovarian cancer samples. **A** comparison of OS between ovarian cancer and controls; **B** comparison of PFS between ovarian cancer and controls

After dividing serous ovarian carcinoma patients into two groups based on the median value, OS and PFS decreased slightly faster in the group with a higher monocyte count (Kaplan–Meier analysis, Fig. 3). A higher MO/LY ratio was significantly correlated with shorter PFS (*p* = 0.001) and OS (*p* = 0.048), which was similar to the results for CA125 (*p* = 0.020 and < 0.001, respectively).

In addition, a higher NE (*p* = 0.029 and 0.014, respectively) and NE% (*p* = 0.011 and 0.004, respectively) significantly predicted shorter OS and PFS times. In contrast, the lower the LY% was (*p* = 0.021 and < 0.001, respectively), the worse the prognosis.

**Fig. 4:**
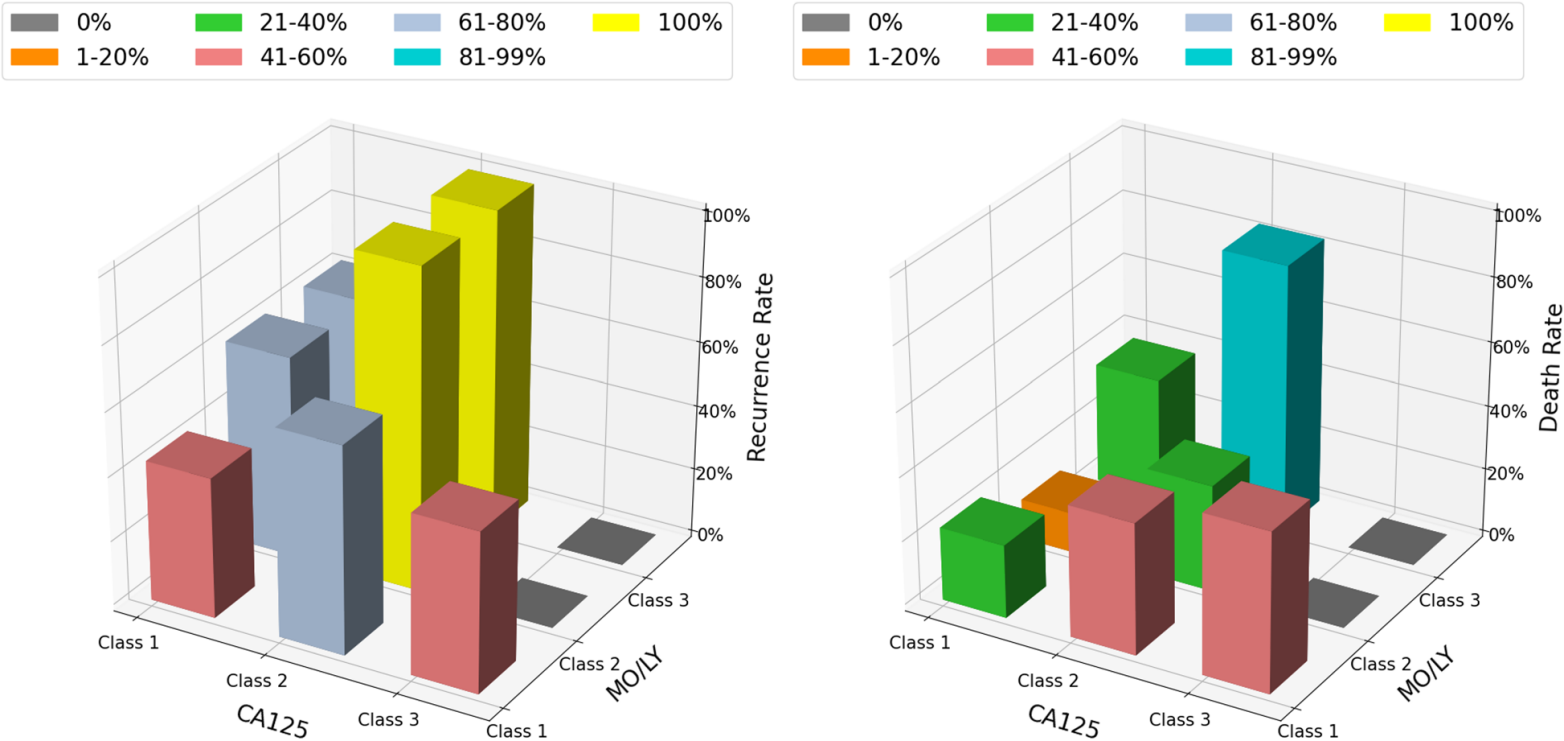
The three-dimensional distribution of CA125, MO/LY and events (death or recurrence). Percentage of patients suffering death (A) or recurrence (B) across tertiles of MO/LY ratio (MO/LY 1–3 = first to third tertiles) and CA125 (CA125 1–3 = first to third tertiles). Graded increases in the risk of death or recurrence are found across increasing tertiles of MO/LY ratio for the first and second tertile of CA125 levels. All patients within the third tertile of CA125 level belonged to the first tertile of MO/LY ratio. Therefore, no cases showed a high MO/LY ratio and high CA125 level at the same time

When assessing death and recurrence according to the tertiles of the MO/LY ratio cross-classified by the tertiles of the CA125 level, both the death rate and recurrence rate increased across the increasing tertiles of the MO/LY ratio for the first and second tertiles of the CA125 level (Fig. 4). All patients within the third tertile of the CA125 level belonged to the first tertile of the MO/LY ratio. Therefore, no cases showed a high MO/LY ratio and high CA125 level at the same time.

### Decision tree to predict survival

**Fig. 5:**
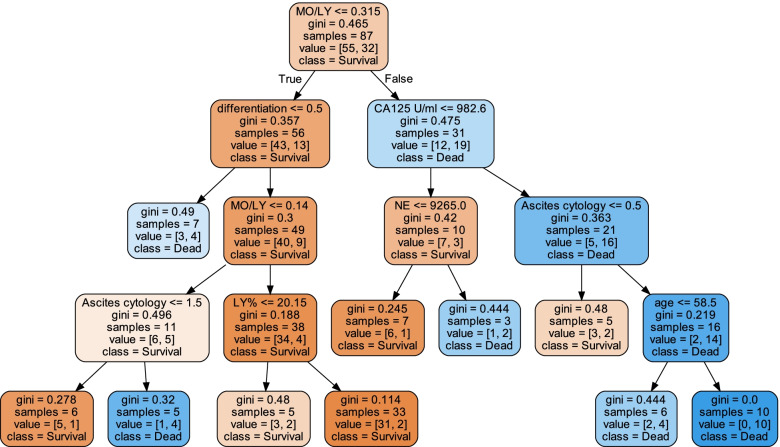
The decision tree visualization for predicting the survival of serous ovarian cancer. In the prediction processing, at the root node, the sample is divided into two groups which have the MO/LY value less or equal to 0.315, or not. Then, the divided samples need to be judged by the second layer leaf node. In the second layer leaf nodes, the value CA125 or differentiation are the standards of classification. After that, it will go through into the third layer of leaf nodes until there is no leaf node left. Finally, when the decision reaches the last leaf node, the survival probability is the number of class samples divide total samples in the node. For example, at the leftmost leaf node, the probability of survival is 5/6 and the probability of death is 1/6

For the decision tree, CA125 was found to be the root node with the largest information gain by using the built-in method of sklearn (Fig. 5). The Gini coefficient reflects the measure of data uncertainty. The smaller the Gini value is, the higher the purity of the potential classes. In each node, the sample number shows the number of samples before being divided, and the value means the number belongs to each class. For example, in the root node, the total number of samples is 42, so the samples are 42. According to whether the CA125 attribute was less than or equal to 3726.05, the samples were split into two groups that contained 35 and 7 samples, respectively.

For prediction processing, at the root node, the sample was divided into two groups based on a CA125 value less than or equal to 3726.05. Then, the divided samples were judged by the second layer leaf node. In the second layer leaf nodes, the value WBC or LNM are the standards of classification. After that, the samples will go through into the third layer of leaf nodes until there is no leaf node left. Finally, when the decision reaches the last leaf node, the survival probability is the number of class samples divided by the total samples in the node. For example, at the leftmost leaf node, the probability of survival is 5/6, and the probability of death is 1/6.

**Fig. 6:**
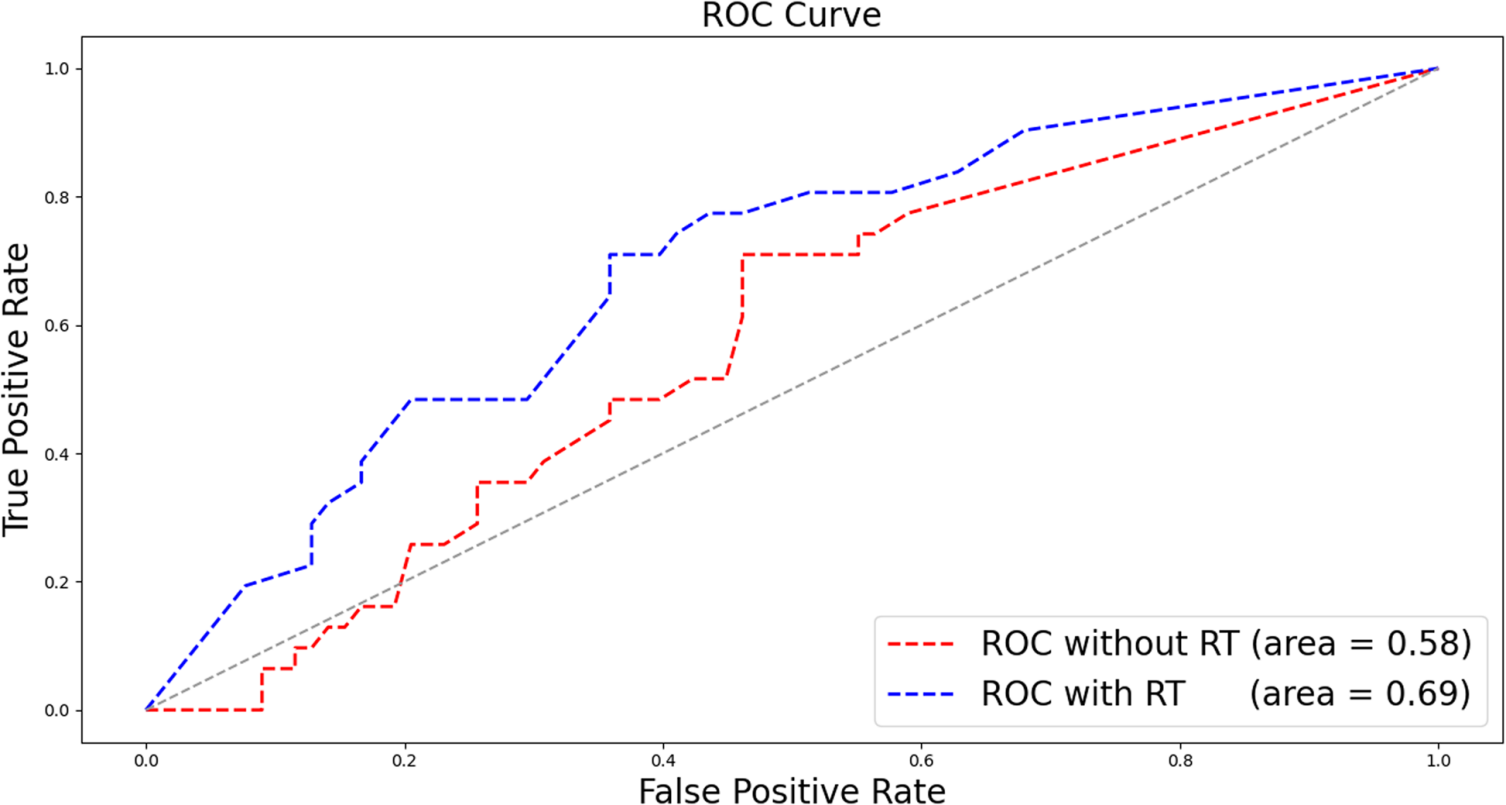
The performance cooperation in ROC curve. The model trained by the features with (blue line) and without blood routine test (RT) (red line)

**Fig. 7:**
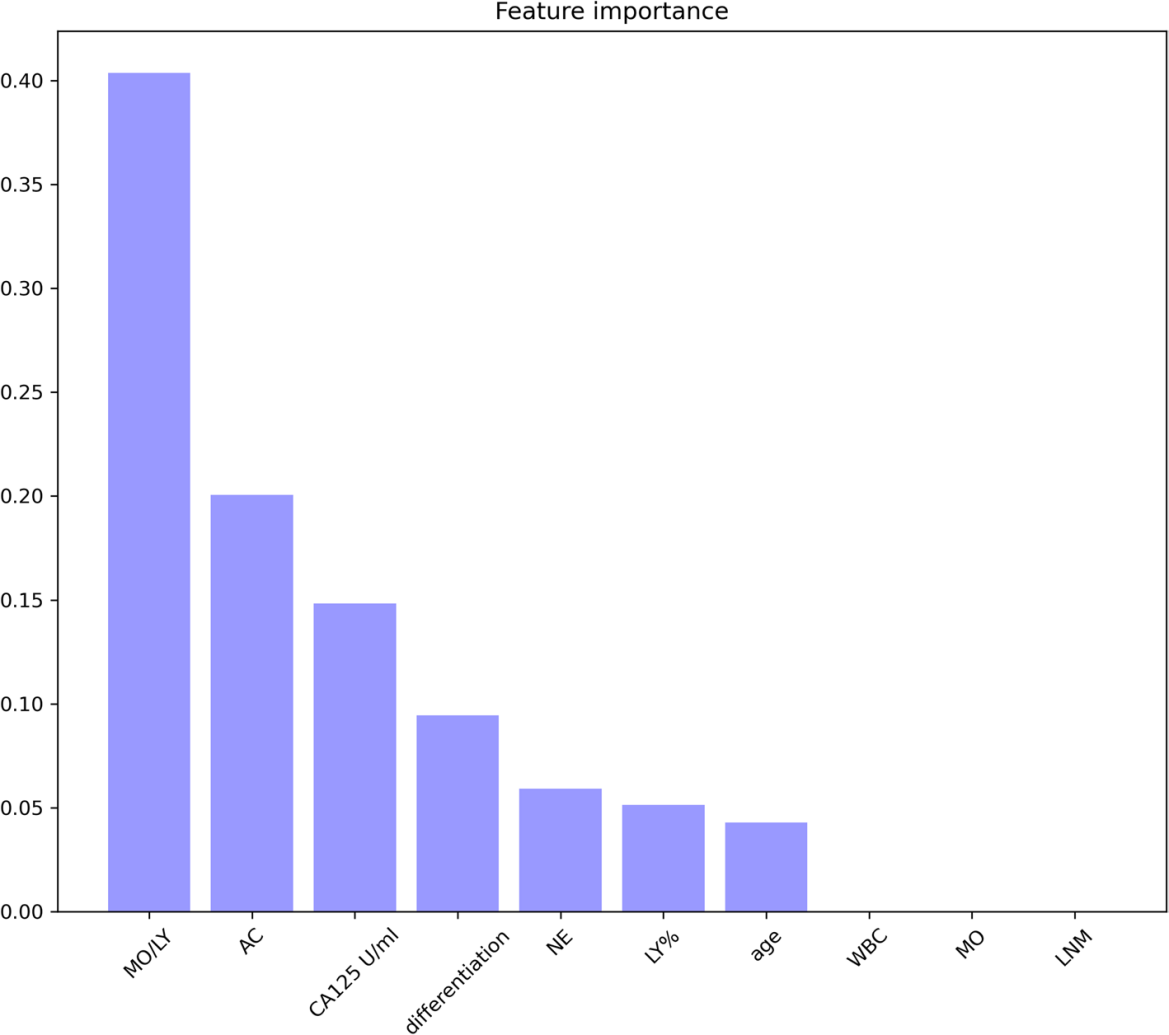
The features importance was shown with the rate of weight distribution of the decision tree

The features involved in the decision tree were the MO/LY, differentiation status, CA125 level, NE, ascites cytology, LY% and age. The survival prediction AUC of the decision tree was 0.69 (95% CI: 0.67–0.70).Meanwhile, the survival prediction AUC of the logsitic regression (LR) was 0.55(95% CI: 0.53–0.57), which means that the performance of decision tree is much bether.The performance cooperation between the model trained by the features with (blue line) and without the routine blood test (RT) (red line) results is shown in Fig. 6. From Fig. 6, it is obvious that the model trained with RT has better performance than that without RT. The feature importance in the decision tree is shown in Fig. 7. The MO/LY, differentiation, CA125, NE, ascites cytology, LY% and age had a high impact, and WBC, MO and LNM had a low impact on the model.

## Discussion

Ovarian carcinoma is the deadliest gynecological carcinoma, and epithelial ovarian cancers are the most common type of ovarian carcinoma. Two-thirds of epithelial ovarian cancers are serous carcinoma [3, 6]. There are some signs that the inflammation caused by pelvic inflammatory disease may be associated with ovarian cancer [9]. Inflammation, cancer immunity and the immune microenvironment often involve various leukocytes [10–12]. In this study, we explored the role of preoperative circulating leukocytes in serous ovarian carcinoma and investigated their value in predicting survival prognosis. We found that most preoperative subtypes of WBCs, including monocytes, neutrophils, lymphocytes, and eosinophils, were significantly different between the serous ovarian carcinoma group and the control group, both in terms of the count and the percentage. These parameters have also been associated with the clinicopathological features of ovarian serous carcinoma.

Monocytes are the largest leukocytes and account for 2–10% of all leukocytes. These cells can migrate from the blood to tissues and then differentiate into macrophages. Monocytes and macrophages perform 3 major roles in the immune system, namely, phagocytosis, antigen presentation and cytokine production [29, 30]. Most macrophages at disease sites are produced via the differentiation of circulating monocytes [31]. Lymphocytes account for 18% to 42% of all circulating leukocytes. Lymphocytes, such as T cells, B cells and natural killer cells, participate in many aspects of the immune response, including cancer immunity [10–12]. Therefore, we also calculated the monocyte-to-lymphocyte (MO/LY) ratio. The results revealed that the preoperative MO/LY was significantly increased in the blood of patients with serous ovarian cancer, similar to the results for monocytes. The higher the ratio is, the worse the prognosis. The possible underlying mechanism may be that monocytes enter the tumor microenvironment and then differentiate into tumor-associated macrophages and promote tumor development [32–34]. Lymphocytes are an important part of the immune response, so when the MO/LY ratio is out of balance, it indicates a poor survival prognosis. It is worth mentioning that the MO/LY seems to show important clinical value, similar to CA125, based on either its predictive value or the results of cross-variable 3D histograms and survival analysis.

In 2012, Vinod Khosla, co-founder of Sun Microsystems, predicted that 80% of clinical work will be replaced by automated machine learning medical diagnostic software in the next 20 years. As an example, in 2020, machine learning technology was used to help diagnose and treat COVID-19 [35]. In this study, we applied a machine learning algorithm to predict the survival outcomes of patients with serous ovarian carcinoma and found that the MO/LY, differentiation status, CA125 level, NE, ascites cytology, LY% and age can be analyzed for survival prediction. This is consistent with the results showing that the MO/LY, CA125 level, NE and LY% are significantly associated with OS and with the NCCN guidelines, which indicate that differentiation, ascites cytology and age are risk factors. In addition, the comparison between the model trained by the features with and without RT shows that the RT has an impact on prediction results. However, when a patient undergoes surgery and/or chemotherapy, the proportion and composition of WBCs in the blood changes significantly, so further research is needed to explore the postoperative situation.

The limitation of this article is mainly the small sample size. In the 3D histogram, there is a lack of data for the MO/LY and CA125, which are both very high. The separate analyses for high-grade and low-grade serous cancers are unable to be performed because of the limitation of the sample size. Therefore, studies on a larger sample size of patients as well as prospective studies are needed. In addition, leukocytes in the blood change according to the state of the body, so the role of postoperative circulating leukocytes still requires much research. Furthermore, the mechanism of action of leukocytes after reaching the tumor tissue site remains unclear.

## Conclusion

The number and percentage of preoperative leukocytes change significantly in patients with ovarian cancer, as well as the MO/LY, and these changes can be correlated with other clinicopathological characteristics, including survival and recurrence. The clinical value of the MO/LY was similar to that of CA125. In addition, the decision trees generated with machine learning can predict the survival of patients with serous ovarian cancer based on the MO/LY, differentiation status, CA125 level, NE, ascites cytology, LY% and age. However, additional research is still warranted.

## Data Availability

The data that support the findings of this study are available from the Beijing Chaoyang Hospital, Capital Medical University but restrictions apply to the availability of these data, which were used under license for the current study, and so are not publicly available. Data are however available from the authors upon reasonable request and with permission of the Beijing Chaoyang Hospital, Capital Medical University.

## Abbreviations

MDSC: Myeloid-derived suppressor cells
WBCs: White blood cells
LMR: Lymphocyte-to-monocyte ratio
EOC: Epithelial ovarian cancer
OS: Overall survival
PFS: Progression-free survival
AI: Artificial intelligence
LNM: Lymph node metastasis
DOD: Recurrence death of disease
FIGO: Federation of Gynecology and Obstetrics
3D: Three-dimensional
PT: Paclitaxel and cisplatin
PAC: Cisplatin + adriamycin + cyclophosphamide
PEI: Cisplatin + etoposide + ifosfamide
MO/LY: Monocytes to lymphocyte
NE: Neutrophils
LY: Lymphocytes
EO: Eosinophils
RT: Blood routine test
